# Determinants of high sensitivity cardiac troponin T elevation in acute ischemic stroke

**DOI:** 10.1186/1471-2377-14-96

**Published:** 2014-05-03

**Authors:** Kashif Waqar Faiz, Bente Thommessen, Gunnar Einvik, Pål Haugar Brekke, Torbjørn Omland, Ole Morten Rønning

**Affiliations:** 1 Institute of Clinical Medicine, University of Oslo, Oslo, Norway; 2 Department of Neurology, Division of Medicine, Akershus University Hospital, Lørenskog N-1478, Norway; 3 Department of Cardiology, Division of Medicine, Akershus University Hospital, Lørenskog, Norway

**Keywords:** High sensitivity cardiac troponin T, Acute ischemic stroke, ECG

## Abstract

**Background:**

A proportion of patients with acute ischemic stroke have elevated cardiac troponin levels and ECG changes suggestive of cardiac injury, but the etiology is unclear. The aims of this study were to assess the frequency of high sensitivity cardiac troponin T (hs-cTnT) elevation, to identify determinants and ECG changes associated with hs-cTnT elevation, to identify patients with myocardial ischemia and to assess the impact of hs-cTnT elevation on in-hospital mortality.

**Methods:**

Patients discharged with a diagnosis of acute ischemic stroke during a 1-year period, were included. Patients diagnosed with acute myocardial infarction (MI) within the last 7 days before admission or during hospitalization were excluded.

**Results:**

In all, 156 (54.4%) of 287 patients had elevated hs-cTnT. The factors independently associated with hs-cTnT elevation were age ≥ 76 years (OR 3.71 [95% CI 2.04-6.75]), previous coronary heart disease (CHD) (OR 2.61 [1.23-5.53]), congestive heart failure (OR 4.26 [1.15-15.82]), diabetes mellitus (OR 4.02 [1.50-10.76]) and lower eGFR (OR 0.97 [0.95-0.98]). Of the 182 patients who had two hs-cTnT measurements, 12 (6.6%) had both a rise or fall of hs-cTnT with at least one elevated value, and ECG manifestations of myocardial ischemia, e.g. meeting the criteria of acute MI. Both dynamic relative change (p = 0.026) and absolute change (p = 0.032) in hs-cTnT were significantly associated with higher in-hospital mortality.

**Conclusions:**

Established CHD and cardiovascular risk factors are associated with hs-cTnT elevation. Acute MI is likely underdiagnosed in acute ischemic stroke patients. Dynamic changes in troponin levels seem to be related to poor short-term prognosis.

## Background

Cardiac troponins are sensitive and specific markers of myocardial necrosis, and are measured routinely in the diagnosis of acute myocardial infarction (MI). The universal definition of acute MI requires detection of rise and/or fall of cardiac biomarker values (preferably cardiac troponin) with at least one value above the 99^th^ percentile of the upper reference limit [1]. The highly sensitive troponin assays meet the analytical criteria required, as they have a limit of detection lower than the 99^th^ percentile of a general population, and a coefficient of variation of 10% or less at this concentration [2]. The National Academy of Clinical Biochemistry has recommended the use of δ 20% in sensitive assays when cardiac troponin is above the 99^th^ percentile [3,4], while Mueller et al [5] have proposed the use of an absolute change of at least 9.2 ng/L. In acute MI, values may remain elevated for two weeks or more following the onset of myocardial necrosis [1].

More widespread use of the highly sensitive assays have led to substantial increase in the detection of elevated levels of cardiac troponin, even among patients admitted for other conditions [6], such as end-stage renal disease (ESRD) [3,7], pulmonary embolism (PE) [8] and epileptic seizures [9]. Several studies have shown elevation of markers of myocardial damage in a proportion of patients with acute stroke [10-13], and elevated levels are associated with mortality [11,13,14]. In a systematic review [15], the prevalence of cardiac troponin elevation in stroke patients was reported to vary from 0 to 34%, but the studies reviewed were heterogeneous and difficult to compare due to different troponin assays and cut-offs. Furthermore, it is uncertain whether this elevation is transient or chronic and whether the etiology is acute myocardial ischemia or comorbidities. In addition, stroke patients often manifest electrocardiography (ECG) changes suggestive of cardiac injury [16,17], but the etiology is unclear.

The aims of the present study were to identify possible determinants associated with high sensitivity cardiac troponin T (hs-cTnT) elevation and to determine the prevalence of ECG changes in patients with acute ischemic stroke. In addition, we wanted to examine whether hs-cTnT values on admission or dynamic changes in hs-cTnT are related to in-hospital mortality.

## Methods

In this retrospective study, all patients aged 18 and above, discharged from Department of Neurology, Akershus University Hospital, with a diagnosis of acute ischemic stroke (ICD-10 I63.x [International Classification of Diseases, 10th revision]) between June 1, 2009 and May 31, 2010, were identified by a systematic search in our clinical database. In the case of multiple admissions during the study period, only the first admission was included. Patients admitted from other hospitals were excluded because of incomplete data. In this patient cohort, we have previously shown admission hs-cTnT levels to be a predictor of all-cause mortality [14].

In total, 367 patients were identified. In the present study, patients were excluded if admission was delayed more than 24 hours after symptom onset (n = 61), if they were diagnosed with acute MI within the last 7 days before admission or during hospitalization (n = 16), or if they were diagnosed with acute PE during hospitalization (n = 3). Patients with a diagnosis of ESRD requiring dialysis were not eligible (n = 0).

Patient characteristics were extracted by chart review and included age, gender and history of cerebrovascular disease (CVD), coronary heart disease (CHD), congestive heart failure, atrial fibrillation, diabetes mellitus, smoking, hypertension and hyperlipidemia. Stroke severity was determined using the National Institutes of Health Stroke Scale (NIHSS) [18]. Values regarding pulse and blood pressure (BP) were registered, and it was recorded whether the patients received intravenous thrombolytic therapy with recombinant tissue plasminogen activator (rt-PA). Stroke etiology was classified according to the Trial of ORG 10172 in Acute Stroke Treatment (TOAST) criteria [19].

Serum concentrations of hs-cTnT were routinely measured on admission and the following morning (up to 24 hours after the first measurement), or earlier at the discretion of the physician on call. The hs-cTnT analyses were performed using a commercially available assay (Roche Diagnostics, Mannheim, Germany). The limit of detection (LoD) was 5 ng/L (pg/mL), and values under this limit were routinely classified as 4.99 ng/L. According to the manufacturer, the upper reference limit (URL; the 99^th^ percentile in a normal reference population) was 14 ng/L. Recently, gender specific 99^th^ percentiles have been published [20]; 13 ng/L in females and 20 ng/L in males.

Biochemical analyses for blood leukocyte count and estimated glomerular filtration rate (eGFR) were performed on admission. eGFR was estimated using the Modification of Diet in Renal Disease (MDRD) formula.

Standard 12-lead ECGs recorded on admission were evaluated by two investigators (GE and PHB), blinded for patient data. Inter-observer differences were resolved by consensus. The following ECG parameters were analyzed: heart rate and rhythm, PR interval, QRS interval, QTc interval, Q wave, ST segment elevation and/ or depression [21], T-wave inversion [21], Sokolow-Lyon voltage [22] and the Cornell product [23]. The definitions of the ECG parameters are presented in Additional file 1: Table S1. Electrocardiographic left ventricular hypertrophy (LVH) was defined as fulfilling the Sokolow-Lyon voltage and/or the Cornell product criteria. Only heart rate and rhythm were evaluated in patients with pacemakers. PR interval was not evaluated in patients with atrial fibrillation/flutter. Sokolow Lyon voltage and the Cornell product were not evaluated in patients with left bundle branch block (LBBB). ST segment elevation, ST segment depression and T-wave inversion were not evaluated in patients with LBBB or LVH.

In-hospital mortality was used as the outcome measure.

### Statistical analysis

Continuous variables are presented as mean values and standard deviation (SD) for normally distributed data and as median values and interquartile range (IQR) for non-normally distributed data. Normality was tested using normality Q-Q plots and Kolmogorov-Smirnov analysis. Categorical variables are presented in absolute values and percentages.

As most variables were not normally distributed, comparisons between groups were made by Mann-Whitney U-test for continuous variables, and Pearson’s χ2-test or Fisher’s exact test for categorical variables (as appropriate). Elevated hs-cTnT was defined as > 14 ng/L (higher than the URL). Spearman’s rank correlation analysis was performed to assess the correlation between hs-cTnT and age and eGFR.

Multivariate logistic regression analysis was performed to identify determinants independently associated with elevated hs-cTnT. Variables associated with hs-cTnT elevation in the univariate analyses with a p-value <0.20 were included in the multivariate analysis. In addition, we performed gender specific multivariate logistic regression analyses using the gender specific 99^th^ percentile values and multivariate linear regression analysis in the entire study population with hs-cTnT as the dependent variable (logarithmically transformed continuous variable because of a non-normal distribution).

Furthermore, multivariate logistic regression analyses were performed to assess whether elevated hs-cTnT on admission or dynamic changes in hs-cTnT were related to in-hospital mortality. Dynamic changes were assessed as i) relative change (percent δ between two measurements with a predefined cut point of 20%); ii) absolute change (δ between two measurements with a predefined cut point of 9.2 ng/L).

Age was dichotomized to higher or lower than the median age in the multivariate analyses.

Associations are presented as odds ratios (OR) with the corresponding 95% CI. A p-value <0.05 was considered to be statistically significant. All statistical analyses were performed using SPSS statistical software version 18 (SPSS Inc, Chicago, IL, USA).

The study was approved by the Regional Committee for Ethics in Medical Research.

## Results

A total of 287 patients were included in the study, with a median age of 76 years (IQR 65-83), and 44.9% were females. Patient characteristics are shown in Table 1. The hs-cTnT concentration on admission was available for all patients, while two hs-cTnT measurements were available for 264 (92.0%) patients. The median hs-cTnT concentration on admission was 15.6 ng/L (IQR 7.7-28.5). Admission hs-cTnT was above the LoD in 249 (86.8%) patients and elevated in 156 (54.4%) patients. Using the gender specific 99^th^ percentile values, hs-cTnT was elevated in 71 (55.0%) females and 65 (41.1%) males.

**Table 1:** Patient characteristics

	**All patients**	**hs-cTnT ≤14 ng/L**	**hs-cTnT > 14 ng/L**	**p value**
	**(n = 287)**	**(n = 131)**	**(n = 156)**	
Age (y)	76 (65–83)	65 (59–76)	81 (74–87)	<0.001
Females	129 (44.9)	59 (45.0)	70 (44.9)	0.977
*Medical history:*				
Cerebrovascular disease	61 (21.3)	18 (13.7)	43 (27.6)	0.004
Coronary heart disease	72 (25.1)	15 (11.5)	57 (36.5)	<0.001
Congestive heart failure	33 (11.5)	4 (3.1)	29 (18.6)	<0.001
Atrial fibrillation	79 (27.5)	20 (15.3)	59 (37.8)	<0.001
Diabetes mellitus	39 (13.6)	10 (7.6)	29 (18.6)	0.007
Smoking	53 (18.5)	36 (27.5)	17 (10.9)	<0.001
Hypertension	179 (62.4)	67 (51.1)	112 (71.8)	<0.001
Hyperlipidemia	86 (30.0)	35 (26.7)	51 (32.7)	0.271
*Vital signs:*				
Pulse	77.4 (±17.3)	76.8 (±15.1)	78.0 (±19.0)	0.818
Systolic BP	165.9 (±30.3)	167.9 (±29.5)	164.3 (±30.9)	0.337
Diastolic BP	87.1 (±15.0)	88.1 (±13.2)	86.2 (±16.3)	0.152
NIHSS	4 (2-9)	3 (1-7)	4 (2-12)	0.001
rt-PA	27 (9.4)	21 (16.0)	6 (3.8)	<0.001
Blood leukocyte count (10^9^/L)	7.6 (6.4-9.8)	7.6 (6.0-9.6)	7.6 (6.6-9.8)	0.276
eGFR (mL/min)	71.0 (±21.8)	79.4 (±19.4)	63.9 (±21.3)	<0.001
*TOAST*				
Small vessel	65 (22.6)	37 (28.2)	28 (17.9)	0.038
Large artery	51 (17.8)	23 (17.6)	28 (17.9)	0.931
Cardioembolic	84 (29.3)	20 (15.3)	64 (41.0)	<0.001
Other/ undetermined	87 (30.3)	51 (38.9)	36 (23.1)	0.004

Categorical variables are presented in absolute values with percentages; n (%). Continuous variables are presented as mean (±SD) or median with interquartile range.

*hs-cTnT*: high sensitivity cardiac troponin T; *BP*: blood pressure; *NIHSS*: National Institute of Health Stroke Scale; *rt-PA*: recombinant tissue plasminogen activator; *eGFR*: estimated glomerular filtration rate; *TOAST*: Trial of ORG 10172 in Acute Stroke Treatment.

Elevation of hs-cTnT in the entire study population was associated with older age (p < 0.001), history of CVD (p = 0.004) and CHD (p < 0.001), congestive heart failure (p < 0.001), atrial fibrillation (p < 0.001), diabetes mellitus (p = 0.007), non-smoking (p < 0.001) and hypertension (p < 0.001) in the univariate analyses. Patients with elevated hs-cTnT had more severe strokes (p = 0.001) and worse renal function (p < 0.001). There were no significant differences regarding pulse and BP.

The median value of the second hs-cTnT concentration was 16.9 ng/L (IQR 9.2-33.4). The median time interval between the two hs-cTnT measurements was 16.0 hours (IQR 10.7-19.8).

Of the 264 patients with serial measurements, 81 (30.7%) patients had dynamic relative change greater than 20%, while 46 (17.4%) patients had absolute change greater than 9.2 ng/L.

The hs-cTnT level was strongly correlated to age (Spearman’s ρ = 0.55, p < 0.001) and eGFR (Spearman’s ρ = - 0.40, p < 0.001).

### ECG

ECGs were available for analysis in 279 (97.2%) patients. Eight ECGs were either unreadable or missing. Additional file 1: Figure S1 shows a flow diagram for the ECG analysis. Three patients (1.1%) had a cardiac pacemaker. Atrial fibrillation/flutter was present in 72 (25.8%) patients, while 63 of 264 (23.9%) patients had LVH (patients with pacemakers and LBBB excluded). Compared to patients with hs-cTnT within the reference limit, patients with elevated hs-cTnT more frequently had atrial fibrillation/flutter (p < 0.001), right bundle branch block (RBBB) (p = 0.026), ST segment depression (p = 0.025), T-wave inversion (p = 0.029) and LVH (p = 0.019). In all, 34 of 276 (12.3%) patients had pathological Q waves, suggestive of prior MI, but there were no significant differences between patients with hs-cTnT within the reference limit and elevation (p = 0.096). The results are summarized in Table 2.

**Table 2:** Findings on admission ECG related to hs-cTnT

	**All patients**	**hs-cTnT ≤14 ng/L**	**hs-cTnT > 14 ng/L**	**p value**
Heart rate (n = 279)	71.9 (±15.5)	73.2 (±16.2) (n = 127)	76.7 (±20.2) (n = 152)	0.296
Pacemaker (n = 279)	3 (1.1)	1/127 (0.8)	2/152 (1.3)	0.996
Atrial fibrillation/ flutter (n = 276) (2)	72 (25.8)	14/127 (11.0)	58/152 (38.2)	<0.001
PR interval (n = 207) (1)	173.4 (±33.0)	167.6 (±31.0) (n = 113)	180.4 (±34.1) (n = 94)	0.005
QRS duration (n = 276) (2)	103.0 (±29.3)	97.7 (±17.1) (n = 126)	107.5 (±35.9) (n = 150)	0.010
QTc interval (n = 276) (2)	416.5 (±38.2)	413.0 (±24.2) (n = 126)	419.5 (±46.7) (n = 150)	0.010
Right bundle branch block (n = 276) (2)	19 (6.9)	4/126 (3.2)	15/150 (10.0)	0.026
Left bundle branch block (n = 276) (2)	12 (4.3)	4/126 (3.2)	8/150 (5.3)	0.381
Q wave (n = 276) (2)	34 (12.3)	11/126 (8.7)	23/150 (15.3)	0.096
Left ventricular hypertrophy (n = 264) (2, 3, 4)	63 (23.9)	21/122 (17.2)	42/142 (29.6)	0.019
ST segment elevation (n = 201) (2, 3, 5)	6 (3.0)	3/101 (3.0)	3/100 (3.0)	0.990
ST segment depression (n = 201) (2, 3, 5)	29 (14.4)	9/101 (8.9)	20/100 20.0)	0.025
T-wave inversion (n = 201) (2, 3, 5)	31 (15.4)	10/101 (9.9)	21/100 (21.0)	0.029

Categorical variables are presented in absolute values with percentages; n (%). Continuous variables are presented as mean (±SD).

*hs-cTnT*, high sensitivity cardiac troponin T.

(1) Excluded if presence of atrial fibrillation/ flutter.

(2) Excluded if presence of pacemaker.

(3) Excluded if presence of left bundle branch block.

(4) Sokolow-Lyon voltage > 3.5 mV and/or Cornell product > 2440 mm x ms.

(5) Excluded if presence of left ventricular hypertrophy.

After excluding patients with pacemakers, LBBB and LVH, a total of 47 of 201 (23.4%) patients had ECG manifestations of myocardial ischemia (ST segment elevation and/ or ST segment depression and/or T-wave inversion). In total, 100 of the 201 (49.8%) patients had elevated admission hs-cTnT, and 29 of 201 (14.4%) had both ECG manifestations of myocardial ischemia and elevated hs-cTnT on admission.

In the subgroup of patients with serial hs-cTnT values, 12 of 182 (6.6%) patients had both ECG manifestations of myocardial ischemia and rise or fall of hs-cTnT (δ ≥ 20%) with at least one value above the 99^th^ percentile of the URL.

### Multivariate analyses

When the factors associated with hs-cTnT elevation in the univariate analyses (p < 0.20) were entered into the multivariate logistic regression analysis, the following factors emerged as significant determinants of elevated hs-cTnT in the entire study population (Table 3): age ≥ 76 years (OR 3.71 [95% CI 2.04-6.75], p < 0.001), previous CHD (OR 2.61 [95% CI 1.23-5.53], p = 0.013), congestive heart failure (OR 4.26 [95% CI 1.15-15.82], p = 0.031), diabetes mellitus (OR 4.02 [95% CI 1.50-10.76], p = 0.006) and lower eGFR (OR 0.97 [95% CI 0.95-0.98], p < 0.001). In the multivariate linear regression analysis (Additional file 1: Table S2), the same five factors were significantly associated with lower hs-cTnT as a continuous variable. In the gender specific multivariate logistic regression analyses (Table 3), age ≥ 76 years and lower eGFR were significantly associated with elevated hs-cTnT concentration in both females and males. In females, hypertension was a statistically significant determinant, while previous CHD, diabetes mellitus and atrial fibrillation emerged as significant determinants in males.

**Table 3:** Determinants of elevated hs-cTnT in acute ischemic stroke, multivariate logistic regression analysis

	**All patients (n = 287),**		**Females (n = 129),**		**Males (n = 158),**	
	**hs-cTnT > 14**		**hs-cTnT > 13**		**hs-cTnT > 20**	
	**p value**	**OR (95% CI)**	**p value**	**OR (95% CI)**	**p value**	**OR (95% CI)**
Age ≥ 76 (median)	**<0.001**	**3.71 (2.04–6.75)**	**0.001**	**5.29 (1.95–14.37)**	**0.001**	**4.11 (1.78–9.54)**
Coronary heart disease	**0.013**	**2.61 (1.23–5.53)**	0.152	2.50 (0.71–8.74)	**0.041**	**2.62 (1.04–6.60)**
Congestive heart failure	**0.031**	**4.26 (1.15–15.82)**	0.591	1.55 (0.32–7.59)	0.077	4.88 (0.84–28.35)
Diabetes mellitus	**0.006**	**4.02 (1.50–10.76)**	0.691	0.75 (0.18–3.18)	**0.005**	**5.31 (1.64–17.16)**
eGFR	**<0.001**	**0.97 (0.95–0.98)**	**0.004**	**0.96 (0.94–0.98)**	**0.013**	**0.98 (0.96–0.99)**
Cerebrovascular disease	0.097	1.90 (0.89–4.10)	0.678	0.78 (0.24–2.53)	0.748	1.18 (0.44–3.15)
Atrial fibrillation	0.459	1.35 (0.61–2.98)	0.997	1.00 (0.29–3.44)	**0.008**	**4.15 (1.45–11.83)**
Smoking	0.459	0.75 (0.34–1.62)	0.552	1.50 (0.40–5.73)	0.282	0.54 (0.18–1.66)
Hypertension	0.221	1.46 (0.80–2.68)	**0.017**	**3.38 (1.24–9.20)**	0.358	0.68 (0.29–1.56)
NIHSS	0.148	1.03 (0.99–1.08)	0.112	1.06 (0.99–1.13)	0.822	0.99 (0.93–1.06)

*eGFR*: estimated glomerular filtration rate; *hs-cTnT*: high sensitivity cardiac troponin T; *NIHSS*: National Institutes of Health Stroke Scale. Significant values in bold.

### In-hospital mortality

In total, 17 (5.9%) patients died in hospital. The median duration of hospitalization was 5 days (IQR 3-8) for these patients. In the subgroup of 264 patients with serial hs-cTnT values, 12 patients (4.5%) died in hospital.

Both dynamic relative change (p = 0.026) and absolute change (p = 0.032) in hs-cTnT were significantly associated with in-hospital mortality (even after adjustment for possible confounders), whereas stable elevation of hs-cTnT was not. The results are summarized in Table 4.

**Table 4:** Association between elevated or change in hs-cTnT and in-hospital mortality, univariate and multivariate logistic regression analysis

	**p value**	**Unadjusted OR (95% CI)**	**P value**	**Adjusted OR (95% CI) (1)**
hs-cTnT > 14 ng/L	0.174	2.10 (0.72–6.12)	0.839	1.15 (0.17–4.22)
log hs-cTnT	0.132	1.51 (0.88–2.59)	0.668	1.21 (0.51–2.89)
δ hs-cTnT ≥ 20% (2)	0.011	4.90 (1.43–16.79)	0.026	5.35 (1.22–23.54)
δ hs-cTnT ≥ 9.2 ng/L (2)	0.007	4.78 (1.53–14.96)	0.032	4.77 (1.15–19.86)

(1) Adjusted for: age ≥ 76 years, gender, stroke severity (NIHSS), coronary heart disease, cerebrovascular disease, atrial fibrillation, smoking, hypertension, diabetes mellitus, eGFR.

(2) Rise or fall with at least one value above the 99^th^ percentile of the upper reference limit (>14 ng/L).

*eGFR*: estimated glomerular filtration rate; *hs-cTnT*: high sensitivity cardiac troponin T; *NIHSS*: National Institutes of Health Stroke Scale.

## Discussion

The principal findings in this study among patients admitted with acute ischemic stroke are: i) hs-cTnT was higher than the URL in 53.4% of the patients; ii) 6.6% of the patients met the criteria of acute MI; iii) patients with elevated hs-cTnT more frequently had ST segment depression and T-wave inversion on ECG, and iv) higher age, history of CHD and congestive heart failure, presence of diabetes mellitus and renal dysfunction were variables significantly associated with hs-cTnT elevation.

Both the median hs-cTnT concentration and the prevalence of detectable hs-cTnT were considerably higher in this population of patients with acute ischemic stroke than previously reported in population-based cohorts and patients with stable CHD [20,24,25]. Previously, it has been demonstrated that elevated hs-cTnT is a predictor of 90-day clinical outcome [26] and long-term mortality [14] in ischemic stroke, thus it is of clinical relevance to identify determinants of elevated hs-cTnT. In a retrospective study, Scheitz et al [27] demonstrated that renal insufficiency, CHD, hypercholesterolemia, higher stroke severity and insular cortex involvement were significantly associated with elevated admission cTnT levels in ischemic stroke patients. A fourth generation assay was used to measure cTnT. In previous studies measuring cardiac troponin with conventional assays in patients with acute stroke, elevated levels have been reported as high as 34% [28]. In a meta-analysis from 2009 by Kerr et al [15], 18% of the patients had elevated troponin level, and these patients were more likely to have ECG changes suggestive of acute MI (OR 3.0). The present study shows that by using a highly sensitive assay with a lower limit of detection, more than 50% of acute ischemic stroke patients have elevated levels of cardiac troponin, even after exclusion of patients diagnosed with acute MI during hospitalization or prior to admission. Interestingly, patients with elevated hs-cTnT more frequently had ST segment depression and T-wave inversion, which are typical ECG manifestations of non-ST elevation MI (NSTEMI). We did not record patient symptoms such as chest pain or shortness of breath, but the presence of hs-cTnT elevation and ECG manifestations of myocardial ischemia indicate that acute MI is likely underdiagnosed in patients with acute ischemic stroke, although the findings are insufficient to conclude whether the ischemia has been caused by type 1 or type 2 MI [1]. Language or cognitive impairments and silent acute MIs before or in concert with the ischemic stroke could be possible explanations. In addition, congestive heart failure and renal failure could be responsible for the troponin elevation.

In a recently published study by Kral et al. [29], 36% of the patients had hs-cTnT higher than the URL, and 22% of the patients presented with elevated hs-cTnT with new or presumed new significant ST-T segment changes or new LBBB.

The etiology of troponin elevation in stroke is not completely understood and still debated. It has been suggested that elevated levels of cardiac markers in acute stroke patients could be related to myocytolysis due to activation of the sympathetic nervous system [30,31]. However, earlier studies have focused on patients with subarachnoidal hemorrhage and arrhythmias. In patients with ischemic stroke, it is difficult to assess whether troponin elevation is due to the acute cerebral injury exerting myocardial stress or to pre-existing comorbidities associated with troponin elevation. Previously, epinephrine [12] and cortisol [28] have been associated with troponin elevation in acute stroke, indicating an imbalance between the parasympathetic and sympathetic nervous system. In our study, there were no significant differences in pulse or BP when comparing patients with normal and elevated hs-cTnT levels. Some studies have highlighted the association between insular cortex involvement and cTnT elevation [27,32], suggesting neurologically induced myocardial injury. We did not assess the possible role of the insular cortex in troponin elevation.

In the study by Scheitz et al. [27], elevated cTnT on admission was an independent predictor of in-hospital mortality, but we could not confirm that a single value obtained on admission was independently associated with short-term outcome However, a novel finding is that dynamic changes in hs-cTnT levels (both relative and absolute change) seem to predict in-hospital mortality, which may indicate that dynamic changes in troponin levels rather than stable elevation related to chronic diseases could be related to poor short-term prognosis.

Our study has certain limitations. First, the retrospective collection of data limits the interpretation of the results, and prospective studies are required to confirm our findings. Second, there was a variation in time between the two hs-cTnT measurements, and the results regarding dynamic changes should therefore be interpreted with caution. It is recommended that blood samples for the measurement of cardiac troponins should be drawn on first assessment and repeated 3–6 hours later [1], while the median time interval was 16 hours in our study. In addition, because of the low number in-hospital mortality events, the results from the multivariate analyses should be considered preliminary, and larger studies are needed to clarify this issue. Future studies should focus on standardized serial measurements of hs-cTnT concentration to determine whether dynamic changes are related to poor short-term prognosis. On the other hand, the strict exclusion criteria excluded patients expected to have elevated troponin levels on admission or during hospitalization.

## Conclusions

We have identified determinants and ECG changes associated with hs-cTnT elevation in patients with acute ischemic stroke, and this study demonstrates a possible role of dynamic changes in hs-cTnT for prediction of in-hospital mortality. Acute MI is likely underdiagnosed in patients with acute ischemic stroke. These results need to be verified in larger prospective studies.

## Competing interests

TO has received speakers’ honoraria from Roche Diagnostics, Siemens Healthcare Diagnostics and Abbott Laboratories. The other authors do not report any potential competing interests. Akershus University Hospital has received research support from Abbott Laboratories.

## Authors’ contributions

Study design: all authors. Data collection: KWF. Statistical analysis: KWF. ECG analysis: GE and PHB. Manuscript preparation: all authors. All authors read and approved the final manuscript.

## Pre-publication history

The pre-publication history for this paper can be accessed here:

http://www.biomedcentral.com/1471-2377/14/96/prepub

## Supplementary Material

Additional file 1: Table S1 Definitions of ECG parameters. **Table S2.** Determinants of high hs-cTnT (logarithmically transformed continuous variable), multivariate linear regression analysis. **Figure S1.** Flow diagram of ECG analysis. Abbreviation list. Click here for file
