# Supplementary material for: Determinants of high sensitivity cardiac troponin T elevation in acute ischemic stroke
Source: BMC Neurol. 2014 May 3;14:96. doi: 10.1186/1471-2377-14-96 (PMC4107722; doi:10.1186/1471-2377-14-96)
Supplement: Additional file 1: Table S1 — Definitions of ECG parameters. Table S2. Determinants of high hs-cTnT (logarithmically transformed continuous variable), multivariate linear regression analysis. Figure S1. Flow diagram of ECG analysis. Abbreviation list. [file 1471-2377-14-96-S1.docx]

**Additional file 1**

**Table S1** Definitions of ECG parameters

| ST segment elevation | ≥0.2 mV in men or ≥0.15 mV in women in leads V2–V3 and/or ≥0.1 mV in other leads |
| --- | --- |
| ST segment depression | ≥0.05 mV in two contiguous leads |
| T-wave inversion | ≥0.1 mV in two contiguous leads with prominent R-wave or R/S ratio >1 |
| Q-wave | ≥0.03 s and ≥0.1 mV deep or QS complex in leads I, II, aVL, aVF, or V4–V6 in any two leads of a contiguous lead grouping (I, aVL,V6; V4–V6; II, III, and aVF) |
| Sokolow-Lyon voltage | Sum of SV1+ RV5 or V6 > 3.5 mV |
| Cornell voltage | Sum of RaVL + SV3 (0.8 mV for female gender) |
| Cornell product | QRS duration x Cornell voltage > 2440 mm x ms |

**Table S2** Determinants of high hs-cTnT (logarithmically transformed continuous variable), multivariate linear regression analysis

|  | B Coefficient | Standard error | t statistic | P value |
| --- | --- | --- | --- | --- |
| Age ≥ 76 (median) | 0.330 | 0.091 | 3.634 | <0.001 |
| Coronary heart disease | 0.237 | 0.100 | 2.361 | 0.019 |
| Congestive heart failure | 0.343 | 0.148 | 2.315 | 0.021 |
| Diabetes mellitus | 0.280 | 0.120 | 2.340 | 0.020 |
| eGFR | -0.009 | 0.002 | -4.568 | <0.001 |

Non-significant factors in the multivariate linear regression analysis: cerebrovascular disease, atrial fibrillation, hypertension, smoking and NIHSS.

hs-cTnT: high sensitivity cardiac troponin T; eGFR: estimated glomerular filtration rate; NIHSS: National Institute of Health Stroke Scale

**Figure S1** Flow diagram of ECG analysis

* Patients with two available high sensitivity cardiac troponin measurements

LBBB: left bundle branch block; LVH: left ventricular hypertrophy

**Abbreviation list**

CHD coronary heart disease

CVD cerebrovascular disease

ECG Electrocardiography

eGFR estimated glomerular filtration rate

ESDR End-stage renal disease

hs-cTnT High sensitivity cardiac troponin T

ICD-10 International Classification of Diseases, 10th revision

IQR Interquartile range

LBBB Left bundle branch block

LoD Limit of detection

LVH Left ventricular hypertrophy

MDRD Modification of Diet in Renal Disease

MI Myocardial infarction

NIHSS National Institutes of Health Stroke Scale

NSTEMI Non-ST elevation MI

PE Pulmonary embolism

rt-PA Recombinant tissue plasminogen activator

TOAST Trial of ORG 10172 in Acute Stroke Treatment

URL Upper reference limit
